# Earwax metabolomics: An innovative pilot metabolic profiling study for assessing metabolic changes in ewes during periparturition period

**DOI:** 10.1371/journal.pone.0183538

**Published:** 2017-08-25

**Authors:** Engy Shokry, Julião Pereira, Jair Gonzalez Marques Júnior, Paulo Henrique Jorge da Cunha, Antônio Dionísio Feitosa Noronha Filho, Jessica Alves da Silva, Maria Clorinda Soares Fioravanti, Anselmo Elcana de Oliveira, Nelson Roberto Antoniosi Filho

**Affiliations:** 1 Laboratório de Métodos de Extração e Separação (LAMES), Instituto de Química (IQ), Universidade Federal de Goiás (UFG), Campus Samambaia, Goiânia, Goiás, Brazil; 2 Escola de Veterinária e Zootecnia, Universidade Federal de Goiás (UFG), Campus Samambaia, Goiânia, Goiás, Brazil; 3 Instituto de Química (IQ), Universidade Federal de Goiás (UFG), Campus Samambaia, Goiânia, Goiás, Brazil; Alexandria University, EGYPT

## Abstract

Important metabolic changes occur during transition period of late pregnancy and early lactation to meet increasing energy demands of the growing fetus and for milk production. The aim of this investigation is to present an innovative and non-invasive tool using ewe earwax sample analysis to assess the metabolic profile in ewes during late pregnancy and early lactation. In this work, earwax samples were collected from 28 healthy Brazilian Santa Inês ewes divided into 3 sub-groups: 9 non-pregnant ewes, 6 pregnant ewes in the last 30 days of gestation, and 13 lactating ewes ≤ 30 days postpartum. Then, a range of metabolites including volatile organic compounds (VOC), amino acids (AA), and minerals were profiled and quantified in the samples by applying headspace gas chromatography/mass spectrometry, high performance liquid chromatography/tandem mass spectrometry, and inductively coupled plasma-optical emission spectrometry, respectively. As evident in our results, significant changes were observed in the metabolite profile of earwax between the studied groups where a remarkable elevation was detected in the levels of non-esterified fatty acids, alcohols, ketones, and hydroxy urea in the VOC profile of samples obtained from pregnant and lactating ewes. Meanwhile, a significant decrease was detected in the levels of 9 minerals and 14 AA including essential AA (leucine, phenyl alanine, lysine, isoleucine, threonine, valine), conditionally essential AA (arginine, glycine, tyrosine, proline, serine), and a non-essential AA (alanine). Multivariate analysis using robust principal component analysis and hierarchical cluster analysis was successfully applied to discriminate the three study groups using the variations of metabolites in the two stress states (pregnancy and lactation) from the healthy non-stress condition. The innovative developed method was successful in evaluating pre- and post-parturient metabolic changes using earwax and can in the future be applied to recognize markers for diagnosis, prevention, and intervention of pregnancy complications in ewes.

## Introduction

Several metabolomic approaches have been conducted for the study of different classes of small-sized metabolites reflecting the key metabolic pathways involved in the transition period between late pregnancy and early lactation in ruminants [1–16]. The majority of these studies utilized traditional biological fluids such as blood, plasma, serum, etc. employing invasive sampling techniques. In this work, we present earwax as an alternative biological matrix for monitoring the changes in the quantitative profiles of a wide range of metabolites during periparturition using targeted and untargeted metabolomics. This could not only help to gain an improved understanding of the metabolic changes occurring during this important period but also in detecting potential derailments which might occur.

Earwax, a waxy substance secreted by ceruminous glands in the ear canal of humans and other mammals, is a mixture of secretions of specialized sweat glands and fatty material from sebaceous glands [17]. Chemically, it is composed of proteins, lipids, glycopeptides, amino acids (AA), short and long chain fatty acids (saturated and unsaturated), aromatic and long chain hydrocarbons, steroids, volatile organic compounds (VOC), and minerals in addition to some environmental pollutants [18].

In humans, it was used as a biological fluid for diagnosis of fungal infections [19] or other pathological conditions as cystic fibrosis, allergic rhinitis, otoschlerosis [20], tumors using certain patterns of sugar, and metabolic diseases [21]. Two metabolic diseases—maple syrup urine disease and alkaptonuria—were identified in earwax before being diagnosed using traditional techniques as blood and urine analysis [22, 23]. Earwax was also used to study lifetime pattern of organic pollutants in a blue whale [24]. In our recent work, earwax was successfully employed in the diagnosis of diabetes in humans as well as discrimination between its types [25]. It has been also applied as an alternative matrix for monitoring of drugs of abuse or drug facilitated crimes [26], exposure to environmental pollutants [27] and in cattle, to detect exposure to fluoroacetate intoxication [28].

Earwax presents a number of advantages in comparison to traditional biological fluids (blood, plasma, serum) as it overcomes the ethical concerns in the invasive sampling techniques involved in sample collection. Thus, it allows acquiring information about an animal without the need for capturing or immobilization which is detrimental and affecting the animal wellbeing. Earwax sampling is also easy and not painful unlike invasive sampling techniques which can pose stress for animals and thus potentially alter the levels of some of the biomarkers detected.

This in addition to merits of earwax over other non-invasive biological matrices (sweat, saliva, hair, urine, feces) including less liability to external contamination as the ear canal is more protected from the external environment, providing sufficient quantities for multiple assays, involving no or minimum pretreatment or time consuming pre-concentration steps, lack of need for vet personnel for sample collection. Moreover, we believe that earwax could serve as an indicator of the cumulative history of physiological changes occurring in the preceding period to the sample collection (up to few weeks) which is the time required for the buildup of the sample [25].

Nevertheless, it presents some limitations as: timely reproduction which makes it not suitable for repeated sampling over short intervals of time due to relatively slow accumulation of earwax (biomarkers are brought into earwax through the blood circulation as it is being secreted and accumulated in the ear), involves complicated instrumentation as gas chromatography/mass spectrometry (GC-MS), liquid chromatography/mass spectrometry (LC-MS) that requires trained personnel, in addition to lack of standardized methods for earwax collection [25, 26]. An overview of the characteristics of earwax in comparison to the other biological matrices is shown in Table 1.

**Table 1:** Characteristics of earwax in comparison to other biological matrices.

Characteristics	Earwax	Invasive biological matrices (Blood, plasma, serum)	Other non-invasive biological matrices				
			Saliva	Sweat	Urine	Feces	Hair
Painful/stressful condition during sample collection	Low	High	Low	Low	Low	Low	Low
Liability to external contamination	Low	Low	High	High	High	High	High
On-site screening possible	No	No	Yes	No	Yes	No	No
Suitability for tracking longterm changes	Yes	No	No	No	No	Yes	Yes
Liability to diurnal variations	No	Yes	Yes	Yes	Yes	No	No
Possibility of repeated sampling over short intervals	No	Yes	Yes	No	Yes	Yes	No
Need for time consuming pre-concentration steps	No	No	Yes	No	Yes	No	No
Liability to blood contamination of samples	No	No	Yes	Yes	Yes	Yes	Yes
Effect of pH on composition	No	Yes	Yes	Yes	Yes	Yes	No
Affected by location of sample collection	No	No	No	Yes	Yes	Yes	Yes (hair position on the body)
Need for vet personnel for sample collection	No	Yes	No	No	Yes	No	No
Analytical costs	Medium	Medium	Medium	High	Medium	Medium	High

## Materials and methods

### Animals, feeding and housing

The trial was conducted in one of the research facilities of the veterinary school of the Federal University of Goiás, Goiânia. A total of 28 healthy Brazilian Santa Inês ewes, aged 2±0.5 years old (average live weight 40 kg), all multiparous were included in this cross-sectional study. Ewes were divided according to their physiological status into 3 subgroups: (1) 9 healthy non-pregnant ewes (HNP); (2) 6 healthy pregnant ewes within the last 30 days of gestation (HP); (3) 13 healthy lactating ewes within 30 days postpartum (HL). All ewes were located in a free stall with straw bedding material with no access to pasture. They were all fed the same basal diet of ground corn grain, soybean meal, sorghum silage, vitamin, mineral supplement, and corn silage. Fresh feed was offered twice a day (0500 and 1600 h). All ewes had free access to water.

### Sample collection

For the experiment, earwax samples were collected directly from both ears of the ewes included in the study using plastic curettes. Sample collection was carried out within 30 days before and after parturition for the pregnant (HP) and the lactating (HL) ewes, respectively. Samples were collected once, all at the same day and timing (1100–1200 h), transferred in Eppendorf tubes which were closed, immediately stored in a freezer at -20°C available at the site of collection, and stored there within 7 days until analyzed.

All procedures performed were in strict accordance with the ethical standards of the ethical committee at the Federal University of Goiás where the study was approved and conducted (Protocol number 027/16).

### Volatile organic compound analysis

Earwax samples (20 mg) were accurately weighed, transferred into 20-mL GC vials and 0.2 μL of 3-methyl cyclohexanone (Polyscience Corp., USA) was added as internal standard (IS). The vials were then tightly sealed with gas tight polytetrafluoroethylene (PTFE)-lined rubber septum caps and analyzed by headspace gas chromatography/mass spectrometry (HS/GC-MS) using Shimadzu GCMS-QP2010 Ultra system and Shimadzu AOC-5000 headspace analyzer (Shimadzu, Japan). The system was equipped with a 2500 μL gas-tight syringe, a VT32-20 tray for 20-mL standard vials (PAL System, Zwingen, Switzerland) plus an additional preheating module LHS0 Combi PAL for six vials with control of temperature and heating time (PAL System, Zwingen, Switzerland). The preheating module parameters were set at: agitation speed (500 rpm), agitation on time (5 s), agitation off time (5 s), incubation temperature (160°C). For the chromatography, an analytical capillary column NST-100 (25 m × 0.25 mm i.d. × 0.3 μm film thickness) (NST, São Paulo, Brazil) with a polyethylene glycol high-polarity stationary phase was used.

The oven temperature was set to 5 min isothermal heating at 30°C, followed by an increase at 2°C/min until 45°C (held for 5 min) then increased by 2°C/min to 50°C (held for 5 min) then at the same rate to 120°C, then increased at 6°C/min to 200°C (held for 5 min) and finally at 5°C/min to 250°C (held for 10 min).

The headspace sampler parameters were: fill volume (2500 μL), fill speed (100 μL/s), injection volume (2500 μL), injection speed (500 μL/s), syringe temperature (150°C), pre-warm time (10 min), equilibration time (5 min), syringe flushing time (1 min), re-flush time (0 ms), and post-flush time (0 ms).

High purity helium (99.999%—5.0) was used as carrier gas with a constant flow rate of 1.0 mL/min. Injector was operated in the splitless mode at 230°C. Start cut-off time for MS recording was 0 min. The MS was operated in electron ionization (EI) mode at 70 eV. Data acquisition was performed in full scan mode from m/z = 40–500 with a scan time of 0.3 s.

Individual constituents were identified in earwax samples by comparing their mass spectra with reference compounds and/or NIST11s Mass Spectral Library and only compounds with ≥ 80% probability of a match to NIST11s library standards were named. Further authentication was achieved by subsequent manual inspection and retention time matching of selected compounds. Quantification of the investigated VOC was based on peak area ratios versus IS.

### Amino acid analysis

#### Preparation of standard solutions

For calibration samples, stock solutions of the reference standards of 18 AA: arginine (Arg), leucine (Leu), phenyl alanine (Phe Ala), lysine (Lys), tryptophan (Trp), alanine (Ala), glutamine (Glu), valine (Val), threonine (Thr), cysteine (Cys), glycine (Gly), tyrosine (Tyr), proline (Pro), isoleucine (Iso-leu), serine (Ser), aspartic acid (Asp), glutamic acid (Glu acid), and citrulline (Citrull) (Sigma Aldrich, USA) were prepared in 0.1% hydrochloric acid, each at a concentration of 2 mg/mL. Subsequent dilutions were made in 0.1% hydrochloric acid to prepare a combined standard solution (1 μg/mL) of the investigated AA. All calibration stocks and working standards were stored at -20°C until use.

#### Sample preparation

Earwax samples (10 mg) were accurately weighed, mixed with 800 μL of 0.1% HCl and subsequently vortexed (IKA MS 3 digital, Sigma Aldrich, USA) for 5 min, then centrifuged in Eppendorf Centrifuge (3000 rpm) (Rotana 460R, Hettich, Germany) for 10 min at 4°C. The supernatant was stored at -20°C until analysis.

Chromatography was carried out using an Agilent 1290 series high performance liquid chromatography (HPLC) system equipped with a quaternary pump and 54 vial plate autosampler (Agilent Technologies, Waldbronn, Germany) operating in gradient mode, coupled with thermostated autosampler and fully controlled by Analyst software (Version 1.5.2). The chromatographic run was performed using a Techsphere silica column (250×4.6 mm, 5 μm) (HPLC Technologies, Cheshire, UK) operated at room temperature. Mobile phase was composed of 3 mM ammonium phosphate in HPLC-grade water containing 0.1% formic acid and 0.1% trifluoroacetic acid (TFA) (Sigma Aldrich, USA) as phase A, HPLC-grade water as phase B, and 100% acetonitrile (J.T. Baker, USA) as phase C. The flow rate was maintained at 1.5 mL/min. The injection volume was 1 μL. Separation was accomplished using a linear gradient from 91% to 60% (phase C) over the first 15 min and then returned to the initial condition from 15 to 17 min and then allowed to equilibrate for 3 min while phase A was maintained at 2% throughout the whole run.

MS measurements were done on API 3200 QTRAP triple quadrupole/linear ion trap mass spectrometer (AB SCIEX, Toronto, Canada) equipped with the TurboIonSpray source fully controlled by the Applied Biosystems/MDS Analyst 1.5.2 software. The instrument was operated in the atmospheric pressure chemical ionization (APCI) in positive mode and MS in multiple reaction monitoring mode (MRM). Ion source parameters were: curtain gas (18 psi), ion source gas 1 (40 psi), ion source gas 2 (0 psi), temperature (450°C), collision gas (CAD) (medium), nebulizing current (NC) (4), interface heater (on).

Quantitative data were acquired using Applied Biosystems/MDS Analyst software version 1.5.2.

#### Validation procedure

Evaluation of method performance including limit of detection (LOD), lower limit of quantitation (LLOQ), linearity, accuracy, precision, carryover, extraction efficiency expressed as recovery (R%), and matrix effect expressed as matrix factor (MF) was performed per ICH guidelines [29].

#### Linearity and lower limit of quantitation

Quantification was based on external calibration method. Calibration samples were prepared at seven different concentrations (each containing the investigated AA). Concentration ranges were chosen based on sensitivities and levels of AA normally encountered in earwax. The linear least-square regression was used to determine the mean intercepts, mean slope and determination coefficients (*R*^*2*^) of the calibration curves.

The LLOQ is defined as the lowest concentration of analyte on the calibration curve with an acceptable accuracy with ±20% (bias) of the nominal value and precision ≤20% (CV%) from six replicates for a predefined dynamic range.

The LOD is defined as the lowest concentration that gives a reproducible instrument response with S/N ratio ≥3.

#### Accuracy and precision

Intraday precision and accuracy were determined by analysing five replicates of the calibrators for all amino acids during a single analytical run. Interday precision and accuracy were determined by analysing three replicates of samples at each level through analytical runs made on five different days.

#### Extraction recovery

The extraction recovery was calculated in terms of R% = C/B x 100, where C is the analyte peak area of blank earwax spiked with a reference standard before the extraction and B is the analyte peak area of earwax spiked with the reference standard after the extraction.

#### Matrix effect

The matrix effect was calculated in terms of matrix factor (MF), obtained from three concentration levels (low, middle and high calibrators), each in triplicate. The MF is defined as the analyte peak area ratio of blank earwax sample extract spiked with an analyte after extraction to the reference standard in 0.1% HCl containing equivalent amount of the analyte neat sample. If MF is equal to 1, this means that no matrix effect is present; if MF <1, this means that there is ionization suppression, whereas if MF >1, this means that there is ionization enhancement.

#### Carryover effect

Carryover was investigated by injecting 1 μL of solvent in triplicate immediately after the highest calibration standard and the response was observed at the retention time of the analyte detected. It should not be >20% of the LLOQ response.

### Analysis of minerals

A total of 28 minerals and elements were tested including (Ag, B, Ba, Be, Ca, Cd, Co, Cr, Cu, Fe, K, Li, Mg, Mn, Mo, Na, Ni, P, Pb, S. Sn, Sr, Ti, U, V, Zn, Se, and I) using an inductively coupled plasma optical emission spectrometer (ICP-OES), model 6300 ICAP Duo (Thermo Fisher Scientific, Massachusetts, USA) with the following accessories: tygon tubes, concentric nebulizer, and cyclonic spray chamber core tube 2 mm.

Samples were used from subjects with the amount of earwax remaining after analysis by HS/GC–MS and high performance liquid chromatography/tandem mass spectrometry (HPLC-MS/MS). Samples (10 mg) were weighed and placed into 15-mL PFA-coated pressure vessels, to which 1 mL of 70% v/v ultrapure grade nitric acid (HNO_3_) (Sigma Aldrich, Brazil), and 1 mL of 30% v/v of ultrapure grade H_2_O_2_ (Vetec^®^ Sigma Aldrich, Brazil) were added. The digestion was carried out in a Provecto Analítica model DGT 100 plus microwave oven (Campinas, Brazil) using the following program of 4 steps: (i) 1 min at 320 W power, (ii) 3 min at 500 W power, (iii) 4 min at 800 W power, and finally (iv) 3 min at 0 W. The resulting solution was diluted to 10 mL using Milli-Q^®^ ultrapure water (Merck KGaA, Darmstadt, Germany) in a 10-mL volumetric flask. The diluted samples were stored at 4°C pending analysis.

The wavelengths were chosen taking into account the lines of highest intensity and the lowest number of interference: B (249.773 nm), Ba (455.403 nm), S (234.861 nm), Bi (223.061 nm), Ca (393.366 nm), Co (228.616 nm), Cr (267.716 nm), Cu (324,754nm), Fe (259.940 nm), K (769.896 nm), Li (670.784 nm), Mg (280.270 nm), Mn (257.610 nm), Mo (202.030 nm), Na (589.592 nm), Ni (231.647 nm), P (178.284 nm), Pb (220.353 nm), Sn (189.989 nm), Mr. (421.552 nm), Ti (337.280 nm), V (292.402 nm), Zn (206.200 nm), Se (196.090 nm), and I (178.276 nm).

The readings of all the elements were performed using the axial configuration of the equipment except Ca and Mg, where the radial configuration was used. The instrumental parameters were optimized to achieve greater emission signal corrected to the trace elements. The parameters used were as follows: Rotation (50 RPM pump), gas flow argon aid (0.5 L/min), pressure the nebulizer argon gas (0.16 Mpa) and power source (1250 Watts).

### Data pretreatment

Following peak deconvolution and integration, GC/MS data were obtained with initial compound annotation conducted by searching against NIST11 library and/or reference standards. Quantitative data set of the AA obtained by HPLC-MS/MS using external calibration was organized in a Microsoft excel spreadsheet in terms of concentration of each AA in μg/L. The quantitative report of each sample generated from ICP–OES was stored as a separate csv format file by Thermo Scientific^™^ Qtegra^™^ Intelligent Scientific Data Solution^™^ software.

The data matrix was then exported to MATLAB R2014b (The MathWorks Natick, USA) and multivariate analysis was performed using PLS Toolbox 8.1 (Eigenvector Research Inc., Manson, USA).

### Statistical analysis

In order to minimize statistical bias resulting from the different magnitudes of the relative compositions of different metabolites, data set derived from GC-MS, HPLC-MS/MS, and ICP-OES was autoscaled prior to multivariate analysis. The intrinsic variations of metabolites in the two stress states (pregnancy and lactation) from the healthy non-stress condition were visualized by robust principal component analysis (RPCA) scores plot and hierarchical cluster analysis (HCA) dendrogram using the furthest neighbor method. Robust instead of classical PCA was employed since outliers can be influential on classical PCA [30]. One-way ANOVA was performed to compare the three groups using a significance level (α) of 0.05.

## Results and discussion

### Alteration of the earwax volatile organic compound metabolome in periparturient ewes

The important VOC in earwax are alcohols, ketones, non-esterified fatty acids (NEFA), and hydroxy urea.

**Fig 1:**
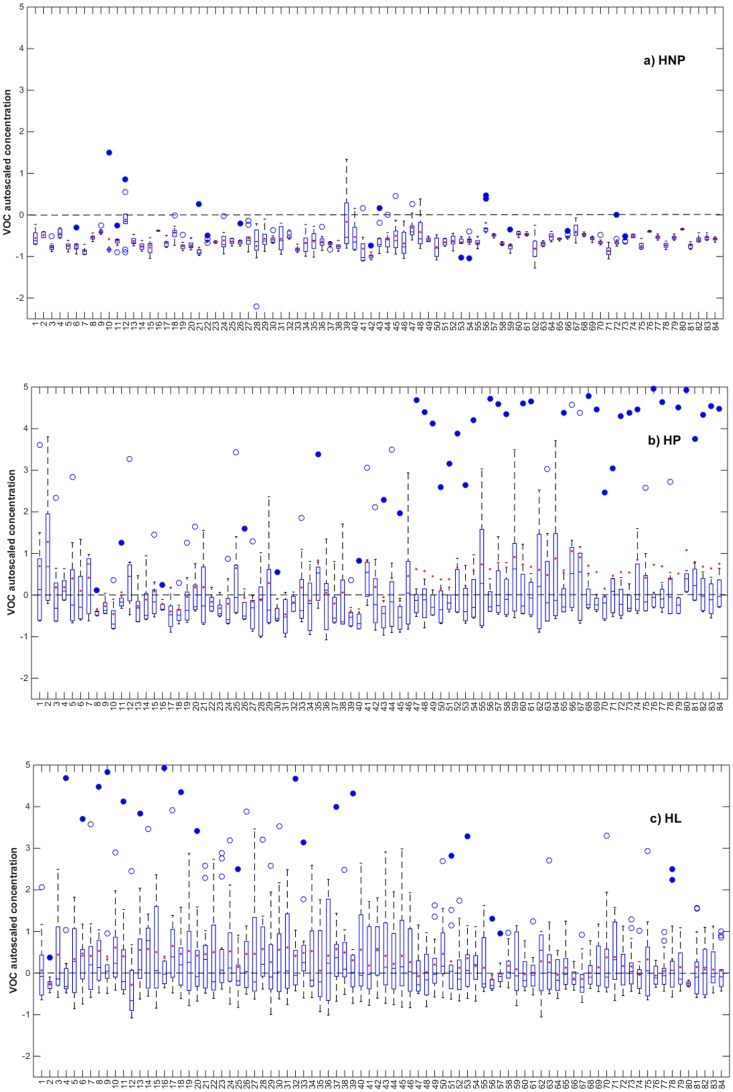
Box-plot diagrams showing autoscaled concentrations of 84 volatile organic compounds (VOC) in earwax of Santa Inês sheep: a) Healthy non-pregnant ewes (HNP), b) Healthy pregnant ewes (HP), and c) Healthy lactating ewes (HL). Unfilled circles (○) represent statistically suspected outliers and filled circles (•) are statistical outliers. VOC numbering is according to Table 2.

Our results show significant elevation in levels of NEFA with different extents in pregnant and lactating ewes including acetic acid (8); decanoic acid (42); tridecanoic acid (57); tetradecanoic acid (58); hexadecanoic acid (66); octadecanoic acid (80) (P<0.05) (Fig 1a–1c). VOC numbering, CAS no., and retention times (Rt) are demonstrated in Table 2.

**Table 2:** Volatile organic compounds (VOC) numbering in Fig 1, its CAS No., and retention times (Rt).

No.	VOC	Rt (min)	CAS No.	No.	VOC	Rt (min)	CAS No.	No.	VOC	Rt (min)	CAS No.
1	Hydroxy urea	1.16	127-07-1	29	Phenol, 4-methyl,	43.04	106-44-5	57	Tridecanoic acid	74.48	638-53-9
2	Ethanol	1.32	64-17-5	30	Phenol, 4-methoxy,	43.22	150-76-5	58	Tetradecanoic acid	75.41	544-63-8
3	Acetone	1.59	67-64-1	31	n-Nonanal	45.06	124-19-6	59	1-Pentadecanol	75.92	629-76-5
4	1,3-Cyclopentadiene	1.97	542-92-7	32	2-Oxo-octanoic acid	50.03	328-51-8	60	1-Hexadecanol	77.89	36653-82-4
5	Propanal, 2-methyl	2.19	78-84-2	33	Phenol, 4-ethyl	50.98	123-07-9	61	1,2-Benzene- dicarboxylic acid, bis (2-methyl- propyl) ester	78.64	84-69-5
6	2-Butanone, 3-methyl	2.63	563-80-4	34	Decanal	53.74	112-31-2	62	Tridecane, 6-methyl-	78.82	13287-21-3
7	2-Butanone	2.76	78-93-3	35	Benzofuran,2,3-dihydro (Coumaran)	55.27	496-16-2	63	1-Heptadecanol	79.15	1454-85-9
8	Acetic acid	4.24	61-19-7	36	(3E)-3-(Amino-methylene) -2H-pyran-2,6(3H)-dione	55.87	20189-42-8	64	2-Nonadecanone	79.87	629-66-3
9	Butanal, 3-methyl,	4.13	590-86-3	37	2(3H)-Furanone, dihydro-5-octyl-(γ-Dodecalactone)	57.52	2305-05-7	65	9-Eicosene	81.46	42448-90-8
10	Butanal, 2-methyl,	4.43	96-17-3	38	1H-indole	60.26	120-72-9	66	n-Hexadecanoic acid	81.64	57-10-3
11	2-Propanone, 1-hydroxy, (Acetol)	4.96	116-09-6	39	Tridecane	61.15	629-50-5	67	Hexadecanoic acid, ethyl ester	82.59	628-97-7
12	2,4-Dimethyl furan	6.57	3710-43-8	40	2-Decen-1-ol	61.41	18409-18-2	68	1-octadecanol	84.03	112-92-5
13	1H-pyrrole, 1-methyl	7.92	96-54-8	41	4-Hydroxy-3-methyl acetophenone	61.65	876-02-8	69	Hexadecane-1,2-diol	84.46	6920-24-7
14	1H-pyrrole	9.02	109-97-7	42	Decanoic acid	64.15	334-48-5	70	n-Nonadecanol-1	84.98	1454-84-8
15	Toluene	9.45	108-88-3	43	Tetradecane	65.86	629-59-4	71	2(3H)-Fuanone, 5-dodecyldihydro- (γ-Palmitolactone)	85.42	730-46-1
16	2-Hexanone	11,13	591-78-6	44	Dodecanal	66.10	112-54-9	72	Cycloeicosane	86.57	296-56-0
17	Acetamide	11.62	60-35-5	45	Oxirane, [(dodecyloxy) methyl]-	68.85	2461-18-9	73	1-Docosene	86.76	1599-67-3
18	Methyl pyrazine	13.53	109-08-0	46	2-Dodecanone	68.95	6175-49-1	74	1-Eicosanol	87.15	629-96-9
19	1H-pyrrole, 2-methyl	15.16	636-41-9	47	Tridecanal	69.39	10486-19-8	75	Hexadecanoic acid, butyl ester	87.39	111-06-8
20	1H-pyrrole, 3-methyl	16.03	616-43-3	48	1-Hexadecene	70.56	629-73-2	76	Cyclodocosane, ethyl-	88,40	0-00-0
21	2-Furanmethanol (Furfuryl alcohol)	17.14	98-00-0	49	Dodecane, 2,6,10-trimethyl-	70.73	3891-98-3	77	Cyclotetracosane	88.81	297-03-0
22	Pyridine, 3-methyl (Picoline)	18.68	108-99-6	50	1-Dodecanol	71.03	112-53-8	78	1-Henicosanol	89.67	15594-90-8
23	2-Heptanone	20.51	110-43-0	51	2-Tetradecanone	71.56	2345-27-9	79	2-Hydroxyhexadecyl butanoate	90.07	77899-01-5
24	Benzaldehyde	28.93	100-52-7	52	Pentadecanal	71.77	2765-11-9	80	Octadecanoic acid	91.36	57-11-4
25	Pyridine, 3,4-dimethyl, (3,4-Lutidine)	31.92	583-58-4	53	1,4-Methano-naphthalene, 6,7-diethyldecahydro-	72.05	16539-02-9	81	Valeric acid, hexadecyl ester	91.84	125164-54-7
26	2-octanone	33.64	111-13-7	54	1,5-Cyclooctadiene, 1-t-butyl-	72.42	0-00-0	82	Octadecanoic acid, butyl ester	92.24	123-95-5
27	Phenol	33.87	108-95-2	55	1-Tetradecanol	73.41	112-72-1	83	Eicosanoic acid, 2-ethyl-2-methyl, methyl ester	92.61	55282-04-7
28	2-Cyclohexene-1-one, 3-methyl (Seudenone)	39.73	1193-18-6	56	Hexadecanal	74.27	629-80-1	84	1-Docosanol (Behenic alcohol)	94.37	661-19-8

This was found in accordance with the fact that ruminants use energy from mobilization of body reserves both in late gestation and during lactation. Fat depots are mobilized, via lipolysis of adipose tissue to increase the energy supply. As a result, NEFA are released into the blood circulation and serve as an important source of energy [31].

A significant increase in the levels of some alcohols was also observed in HP and HL principally ethanol (2) (P<0.0005), in addition to furfuryl alcohol (21), 1-dodecanol (50), 1-tetradecanol (55), 1-pentadecanol (59), 1-heptadecanol (63), 1-nonadecanol (70), eicosanol (74), heneicosanol (78), and docosanol (84) with (P<0.05) (Fig 1a–1c). In the literature, pregnant sheep were already used as an experimental model to monitor the effect of alcohol exposure on the maternal metabolism [32]. Thus, the developed method being able to detect alcohols can be applied as a simple method for monitoring alcohol exposure using earwax.

Regarding ketones, all of the ketones detected in earwax samples of HP and HL showed significant increase in their levels in comparison to HNP (P<0.05) with the exception of 2-hexanone (16). These ketones include: acetone (3), 2-butanone, 3-methyl (6), 2-butanone (7), 2-heptanone (23), 2-octanone (26), cyclohexene-1-one 3-methyl, (28), 3E)-3-(Aminomethylene)-2H-pyran-2,6(3H)-dione (36), 4-hydroxy-3-methyl acetophenone (41), 2-dodecanone (46), 2-tetradecanone (51), and 2-nonadecanone (64) (Fig 1a–1c). This also relates the levels of ketones to the negative energy imbalance occurring during pregnancy and lactation. There are also studies confirming the increase in the levels of ketone bodies (acetone, acetoacetate and β-hydroxybutyrate) in other biological fluids such as milk and blood of subclinical ketotic pregnant cows and ketotic dairy cows [33, 34] occurring as a result of negative energy balance.

Hydroxy urea (1), a normal volatile component in earwax of ewes, showed an increase in both HP and HL than in HNP ewes. In the literature, no reports on hydroxyurea were found but there were studies on plasma urea levels [35] showing that it starts increasing in pregnant Barki ewes from the 10^th^ week of pregnancy, reaching the maximum at parturition. Same results were reported by Durak and Altinek [16]. This could be explained that the glomerular filtration and urea clearance in ewes decrease in late pregnancy and lactation [36]. Another explanation of the increase in the level of urea, could be attributed to the negative energy balance as a result of metabolic stress suffered during pregnancy and lactation [37, 38].

In other studies, level of urea was found slightly higher in pregnancy than that of early lactation but it was highest at 55 days lactation [39].

### Alteration of the earwax amino acid metabolome in periparturient ewes

The change in AA composition was quantified using ion-pair chromatography coupled with MS/MS detection benefiting of the zwitterionic character of the AA. The positive charged amino group is bounded by the anionic ion pair reagent (TFA). The method was validated and the assay validation parameters results are displayed in Table 3.

**Table 3:** Results of assay validation parameters of the HPLC-MS/MS method for the analysis of amino acids (AA) in earwax of Santa Inês sheep.

AA	MRM transitions	Linearity μg/L	LOD μg/L	LOQ μg/L	Precision					R% ±(CV%)	MF ±SD	Carry over (%)
					Nominal Concentrations μg/L	Intraday (5 replicates)		Interday (In triplicate for 5 days)				
						Accuracy (%)	CV (%)	Accuracy (%)	CV (%)			
Arg	175.3>70.0	6.0–100.0	1.8	6.0	6	101.1	2.63	97.8	7.00	90.2±0.63	1.08±0.07	0.5
					50	106.2	4.19	83.5	7.38			
					100	94.8	14.98	91.2	4.24			
Leu	132.2>86.3	4.0–100.0	1.2	4.0	4	97.5	7.96	94.8	3.40	87.2±0.52	0.84±0.04	1.6
					50	108.3	8.06	115.7	5.76			
					100	93.6	2.27	98.0	4.10			
Phe Ala	166.4>120.0	4.0–100.0	1.2	4.0	4	94.2	4.06	95.9	1.74	86.3±3.65	1.00±0.12	1.3
					50	88.4	6.24	107.1	15.15			
					100	90.3	7.36	90.5	5.14			
Lys	147.3>56.2	1.0–100.0	0.3	1.0	1	95.1	17.99	110.9	12.46	89.0±0.52	1.05±0.13	1.6
					50	90.3	14.71	85.1	6.13			
					100	95.8	6.03	97.1	13.63			
Trp	205.4>146.2	1.0–100.0	0.3	1.0	1	89.0	19.02	100.6	12.82	89.5±2.25	0.94±0.07	2.0
					50	97.2	0.58	98.5	3.15			
					100	87.5	0.97	87.8	2.44			
Ala	90.1>44.2	1.0–100.0	0.3	1.0	1	79.3	5.80	83.6	11.34	87.6±1.62	0.94±0.12	1.9
					50	99.0	6.93	101.5	9.05			
					100	93.4	4.10	94.8	4.35			
Glu	147.2>84.2	1.0–100.0	0.3	1.0	1	98.4	6.65	104.5	7.27	90.9±1.21	0.94±0.07	1.2
					50	91.1	8.26	92.2	13.20			
					100	102.0	2.99	99.8	9.86			
Val	118.3>72.3	4.0–100.0	1.2	4.0	4	83.6	8.65	92.6	10.71	84.8±1.02	1.00±0.15	1.3
					50	118.0	8.39	109.9	12.39			
					100	101.0	1.04	101.1	8.72			
Thr	120.2>74.3	4.0–100.0	1.2	4.0	4	87.0	4.99	88.3	1.23	81.1±0.01	0.81±0.05	1.8
					50	93.1	6.45	99.7	6.65			
					100	98.1	0.72	94.5	3.35			
Cys	241.3>152.1	1.0–100.0	0.3	1.0	1	103.0	7.92	111.9	7.60	96.8±0.05	1.12±0.03	0.5
					50	97.7	7.55	88.4	7.24			
					100	88.5	5.06	105.1	15.06			
Gly	76.0>30.3	1.0–100.0	0.3	1.0	1	95.5	2.22	91.5	5.01	85.3±3.21	0.97±0.16	0.7
					50	117.7	13.18	108.2	13.77			
					100	89.7	5.48	99.9	12.40			
Tyr	182.4>136.3	4.0–100	1.2	4.0	4	94.7	6.89	90.0	6.95	84.1±1.87	0.92±0.10	3.6
					50	94.4	12.51	108.9	12.07			
					100	87.6	9.63	91.2	3.40			
Pro	116.2>70.2	1.0–100.0	0.3	1.0	1	77.8	4.09	85.3	10.42	89.50±0.30	0.83±0.01	1.25
					50	106.0	3.77	102.6	9.24			
					100	101.6	4.97	102.0	4.10			
Iso-leu	132.4>86.2	4.0–100.0	1.2	4.0	4	86.1	10.08	86.6	2.23	89.8±0.10	0.84±0.01	1.5
					50	117.7	2.60	108.6	7.69			
					100	100.5	3.01	95.7	7.60			
Ser	106.0>60.0	4.0–100.0	1.2	4.0	4	89.3	7.58	88.7	3.93	78.3±0.25	0.95±0.18	1.7
					50	103.9	9.32	105.6	4.55			
					100	92.8	1.49	93.4	6.11			
Asp	134.1>73.9	1.0–100.0	0.3	1.0	1	86.0	15.73	89.3	7.35	81.6±1.06	0.95±0.18	1.6
					50	106.7	3.79	106.5	13.00			
					100	88.1	16.19	99.3	12.30			
Glu acid	148.3>84.2	1.0–100.0	0.3	1.0	1	83.7	0.69	90.7	6.84	90.2±0.71	0.80±0.06	1.3
					50	92.4	7.22	97.8	13.06			
					100	95.2	5.58	100.2	15.57			
Citrull	176.3>70.0	1.0–100.0	0.3	1.0	1	110.9	11.39	107.4	3.64	89.8±0.26	1.07±0.02	7.6
					50	93.5	4.71	93.8	9.22			
					100	95.6	4.89	95.1	9.34			

**Fig 2:**
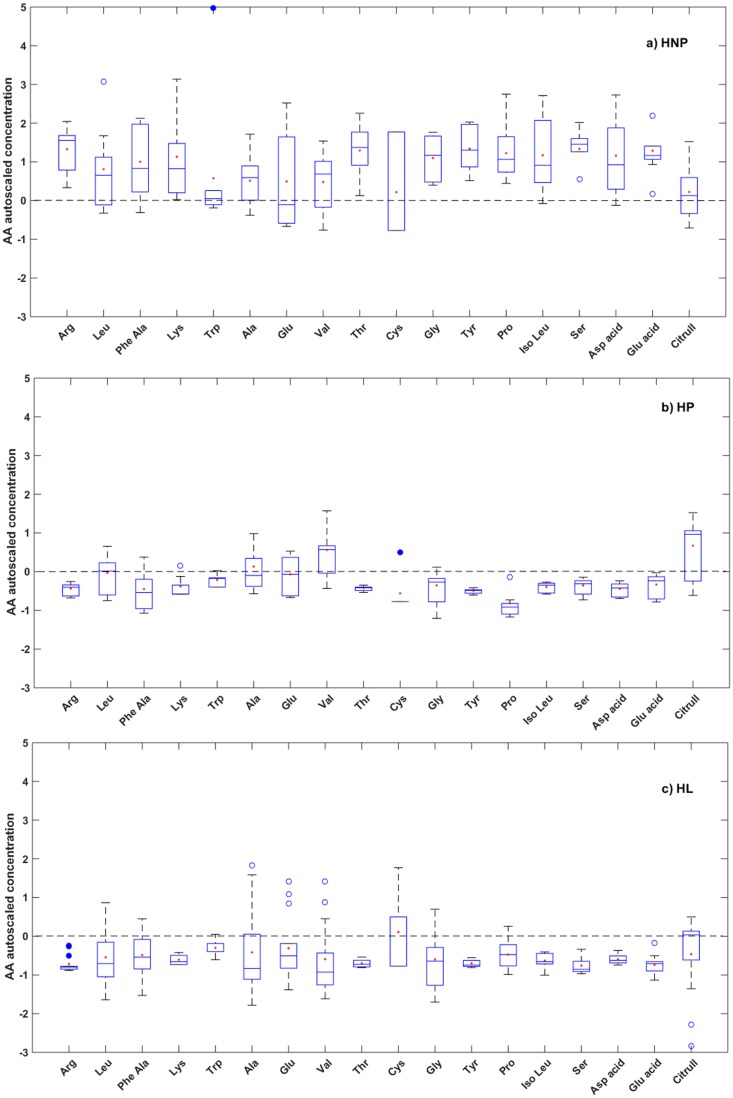
Box-plot diagrams showing autoscaled concentrations of 18 amino acid metabolites (AA) in earwax of Santa Inês sheep: a) Healthy non-pregnant ewes (HNP), b) Healthy pregnant ewes (HP), and c) Healthy lactating ewes (HL). Unfilled circles (○) represent statistically suspected outliers and filled circles (•) are statistical outliers.

According to our results, there are 18 AA in earwax, of which 14 show significant variations in response to physiological changes during periparturition as Arg, Lys, Thr, Gly, Tyr, Pro, Iso Leu, Ser, Asp acid, Glu acid (P<0.0001), and to a lesser extent Phe Ala (P<0.0005), Leu (P<0.005), Val (P<0.01), and Citrull (P<0.05) (Fig 2a–2c). In the overall, HNP AA levels (Fig 2a) are higher than AA levels for both HP (Fig 2b) and HL (Fig 2c). The results indicate variations in essential AA (Leu, Phe Ala, Lys, Iso Leu, Thr, Val) in accordance with results reported [40], some conditionally essential AA (Arg, Gly, Tyr, Pro, Ser), and minor variation in Ala, a non-essential AA principally in HL group. For conditionally essential AA, their synthesis can be limited under special physiological/pathophysiological conditions, such as catabolic distress such as in pregnancy and lactation [41].

This can be explained that during pregnancy, AA are actively transported across the placenta to be utilized by the fetus for protein synthesis and tissue growth. They are also oxidized as an energy source particularly in underfed ewes where the supply of glucose to the fetus is limited [42], especially that glucose and AA are known to be the main energetic substrates for fetal development and colostrum/milk production [43].

It should be also considered that during pregnancy, even if the energy intake is adequate for sufficient total protein production, the supply of certain essential AA may not be enough in relation to the needs of the fetus [44]. Moreover, there were reports on the reduction in the serum AA concentration due to increased maternal blood volume during gestation [45] which, in turn, is caused by increase in serum estrogen levels [46].

As for lactation, some of the AA available from feed are used for gluconeogenesis when energy is not adequate as well as for energy production [47].

### Alteration of the earwax minerals in periparturient ewes

**Fig 3:**
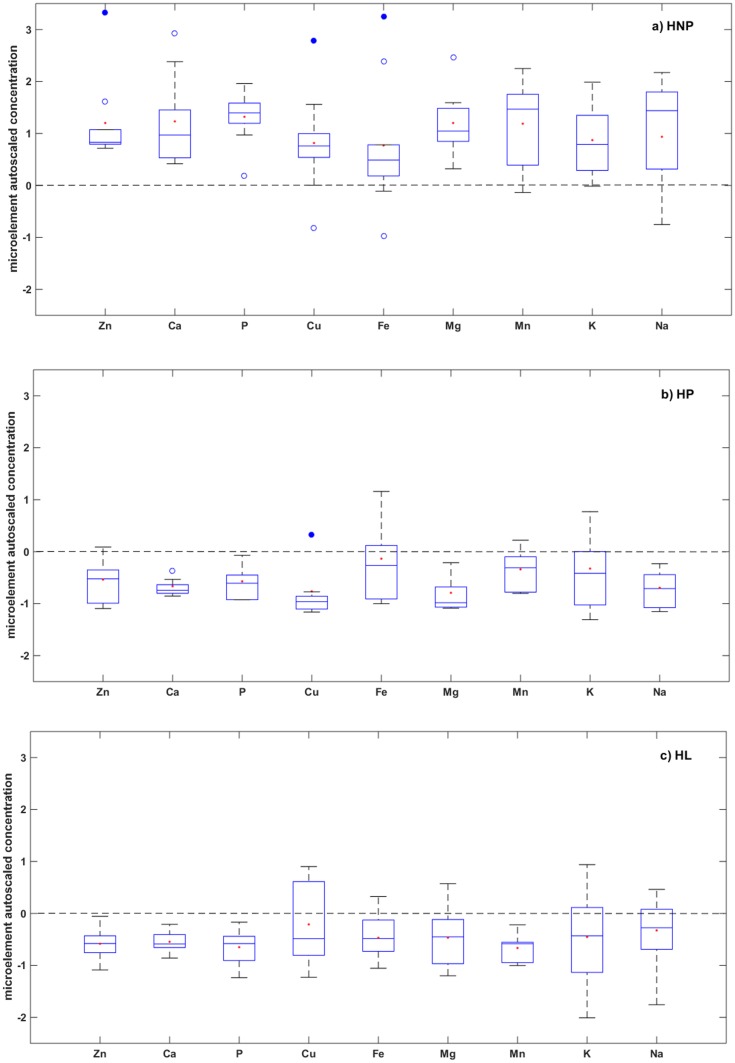
Box-plot diagrams showing autoscaled concentrations of 9 trace elements in earwax of Santa Inês sheep: a) Healthy non-pregnant ewes (HNP), b) Healthy pregnant ewes (HP), and c) Healthy lactating ewes (HL). Unfilled circles (○) represent statistically suspected outliers and filled circles (•) are statistical outliers.

From the 28 elements tested in earwax samples, only 9 were successfully detected and quantified namely (Zn, Ca, P, Cu, Fe, Mg, Mn, Na, and K). They all showed a decline in their concentration in periparturient periods (P<0.005) (Fig 3a–3c).

Regarding Ca levels in earwax, they significantly decrease in pre- and postparturient samples (P<0.0001) matching results on Ca levels in plasma and serum presented in earlier studies [48], where it was reported that Ca levels markedly decrease during late pregnancy, reaching minimum levels at parturition, then continued to decrease for the first few weeks postpartum, ultimately returning to the level before pregnancy [45]. This could be attributed to increased demand for Ca for mineralization of fetal skeleton.

Same results were reported on blood Ca concentrations in ruminants with clinical and subclinical ketosis or pregnancy toxemia due to mineral deficiencies that typically result from ketosis due to disturbances in feed consumption [49].

Meanwhile, results obtained on Na, K, and Mg concentrations showed that they all decreased significantly in HP and HL in agreement with results previously reported on the same minerals in blood, where erythrocytic Ca, Mg, Na, and K concentrations were found to decrease during the last three weeks of pregnancy, day of parturition, and remained low 2 weeks postpartum as a result of loss of these ions through the colostrum [45].

In other reports, serum Na levels were low before and at parturition but started to increase after parturition being highest at weeks 3 and 4 postpartum however, returning to levels at parturition by week 5. By the contrary, Dakka and Abd El All [49] showed that serum Na level increased during late pregnancy in sheep.

With regard to P, it was found to decrease significantly in both groups HP and HL (P<0.0001). In the literature, there were studies confirming no significant differences in plasma P concentrations between different physiological stages [50] while others showed significant decrease of plasma P levels during pregnancy in ewes [51].

This, to an extent, showed similarity of our results obtained using earwax and other biological fluids such as serum and plasma.

### Multivariate analysis

**Fig 4:**
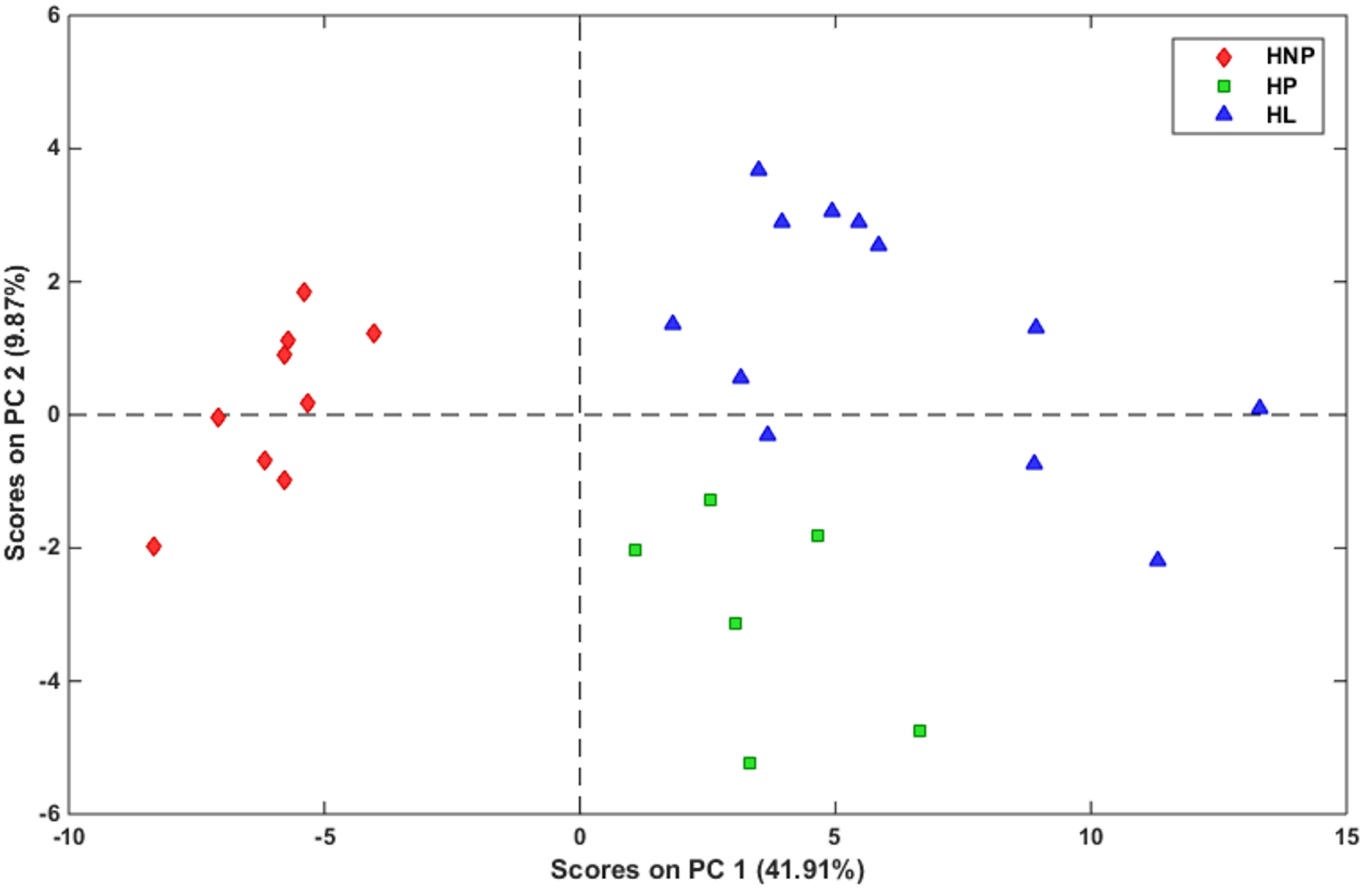
RPCA score plot of 28 earwax samples from Santa Inês sheep using 111 metabolite signals analyzed by HS-GC/MS, HPLC-MS/MS, and ICP-OES: a) Healthy non-pregnant ewes (HNP) (♦), b) Healthy pregnant ewes (HP) (■), and c) Healthy lactating ewes (HL) (▲).

**Fig 5:**
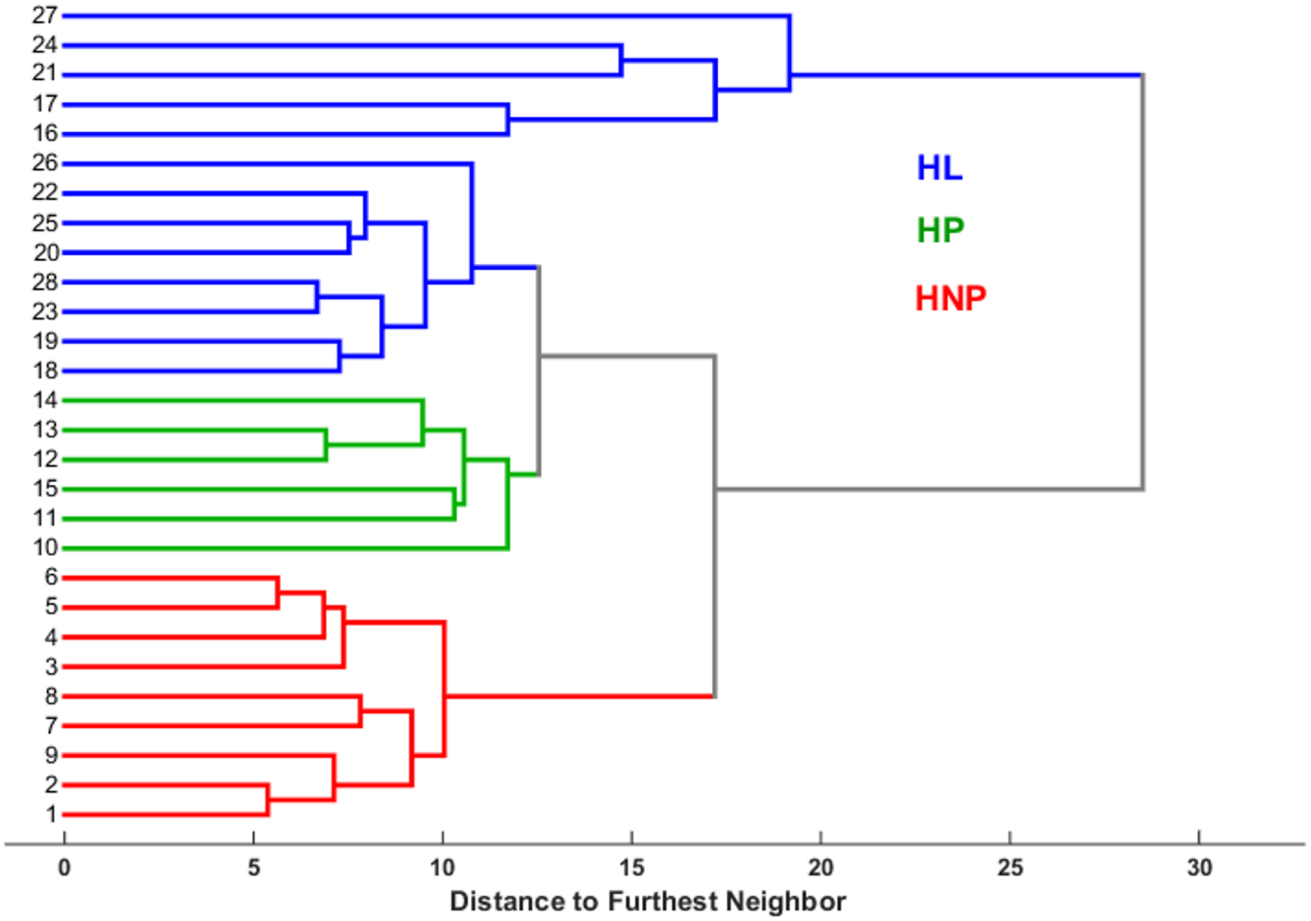
Dendrogram of hierarchial cluster analysis (HCA) of 28 earwax samples from Santa Inês sheep using 111 metabolite signals analyzed by HS-GC/MS, HPLC-MS/MS, and ICP-OES: a) Healthy non-pregnant ewes (HNP) (red), b) Healthy pregnant ewes (HP) (green), and c) Healthy lactating ewes (HL) (blue).

To relate physiological changes associated with pregnancy and lactation with changes in the metabolome, RPCA of marker compounds (metabolites and micronutrients) was performed on the analytical data obtained from HS-GC/MS, HPLC-MS/MS, and ICP-OES of all samples. Fig 4 shows the score plot of the first two principal components (PCs) that explains 51.78% of the total data variance. This plot indicates a clear distinction between the three groups: HNP, HP, and HL. As observed, the HNP samples (left side of Fig 4) could be discriminated from the other two groups (HP and HL) using PC1 alone, as they are concentrated on the negative side of PC1. Meanwhile, PC2 was able to discriminate to a great extent between the HP and HL located on negative and positive sides of PC2, respectively with just few exceptions where 3 HL samples were grouped together with HP group. As seen, HL ewes showed greater heterogeneity than the other two groups studied due to the variation in the metabolite composition among the days during lactation in agreement with the results in the literature indicating significant variations in the metabolite compositions in plasma, serum, and other biological fluids among different days during lactation period in ewes [52]. The sample heterogeneity found in HL group justifies using RPCA instead of the classical PCA. This separation was further confirmed using HCA (Fig 5).

**Fig 6:**
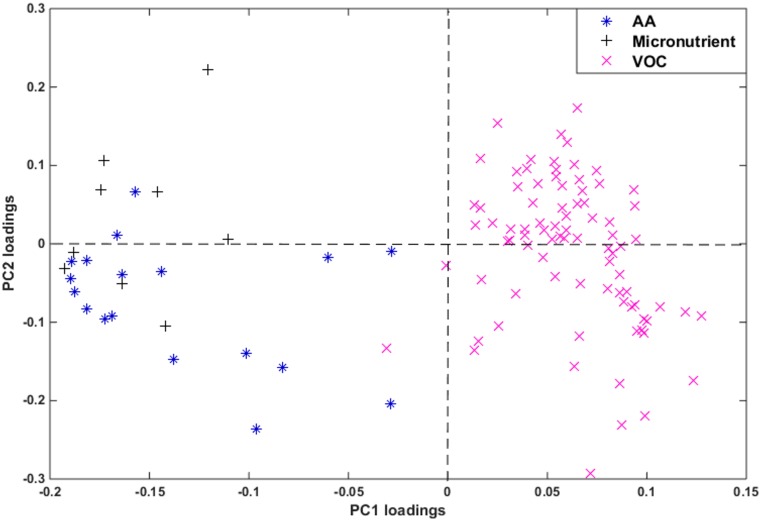
RPCA loadings plot of 111 metabolite signals analyzed by HS-GC/MS, HPLC-MS/MS, and ICP-OES: Amino acids (AA, *), minerals (+), and volatile organic compounds (VOC, ×).

As seen in the dendrogram of Fig 5, clear distinction was obtained between the three groups using HCA. Fig 6 shows the RPCA loadings plot. Two major groups of metabolites emerged from this analysis, corresponding to compounds that either accumulate or decline in response to these physiological changes. The AA (* symbols) and micronutrients (+ symbols) were found to influence most HNP ewes while the VOC (× symbols) influence most the other two groups (HP and HL). This can be confirmed after comparing Figs 4 and 6.

It should be put into consideration that even though RPCA and HCA methods were successfully applied in the current work, these results are only indications of grouping patterns considering the limited number of samples analyzed.

Based on our results, we believe that that metabolomics using earwax as a biological matrix reflects the metabolic changes associated with periparturition. The present approach involved monitoring a wide range of metabolites (fatty acids, alcohols, ketones, amino acids and minerals) using mass spectrometry-based metabolomics (MS-based metabolomics) and ICP-OES. This could be advantageous as the biological complexity of the agricultural animals unavoidably requires a system biology approach that can study the complex interactions in the biological systems using a method of integration and not reduction [53].

Generally, MS-based metabolomics has this power being able to simultaneously analyse several hundreds of metabolites in a biological sample, which are the functional end products of the biological processes [54]. Thus, it allows to differentiate individual phenotypes better than with conventional clinical end points or with small set of metabolites and consequently exploring the physiological status in a more global way [55]. It also presents high sensitivity, high throughput, better identification power for unknown molecules in samples (i.e. by using mass spectral libraries), greater simplicity in handling complex samples by eliminating or minimizing the sample work up, preseparation and chemical manipulation being coupled to separation techniques such as LC and GC [56]. In the same time, it does not suffer the problems related to cross reactivity where high compound specificity is achieved by combined separation and spectral identification which makes it highly sensitive even for minor changes [57]. Moreover, there is no need to develop different methods for different metabolites as in many commercial assays [57]. That is why the applications of MS-based metabolomic studies both targeted and untargeted in the field of animal production and nutrition research are rapidly growing [58].

The earwax is also a promising alternative biological matrix being not only non-invasive but also overcoming several disadvantages faced in other biological fluids such as easy collection, no diurnal variations (results not shown), less liability to external contamination, minimum or no sample pre-concentration or pretreatment, no need for vet personnel for sample collection, and ability for relatively long term monitoring (e.g. few weeks) required for sample build-up, where the biomarkers reach the earwax through the blood circulation as it is being secreted and accumulated in the ear [27]. On the other hand, the study of some important parameters that may influence the application of earwax as a diagnostic biological matrix in animals such as effect of grazing, diet, quantity and quality of earwax in the same breed, etc.) should be addressed in future studies by the authors.

In conclusion, we believe that combining advantages of metabolomics with non-invasive sampling using earwax could hold a great promise in pregnancy and lactation research keeping in mind the technical and economic challenges.

Based on our results, we believe that that metabolomics using earwax as a biological matrix reflects the metabolic changes associated with periparturition. The present approach involved monitoring a wide range of metabolites (fatty acids, alcohols, ketones, amino acids and minerals) using mass spectrometry-based metabolomics and ICP-OES. This could be advantageous as the biological complexity of the agricultural animals unavoidably requires a system biology approach that can study the complex interactions in the biological systems using a method of integration and not reduction [53].

Generally, MS-based metabolomics has this power being able to simultaneously analyse several hundreds of metabolites in a biological sample, which are the functional end products of the biological processes [54]. Thus, it allows to differentiate individual phenotypes better than with conventional clinical end points or with small set of metabolites and consequently exploring the physiological status in a more global way [55]. It also presents high sensitivity, high throughput, better identification power for unknown molecules in samples (i.e. by using libraries), greater simplicity in handling complex samples by eliminating or minimizing the sample work up, preseparation and chemical manipulation being coupled to separation techniques such as LC and GC [56]. In the same time, it does not suffer the problems related to cross reactivity where high compound specificity is achieved by combined separation and spectral identification which makes it highly sensitive even for minor changes [57]. Moreover, there is no need to develop different methods for different metabolites as in many commercial assays [57]. That is why the applications of MS-based metabolomic studies both targeted and untargeted in the field of animal production and nutrition research are rapidly growing [58].

The earwax is also a promising alternative biological matrix being not only non-invasive but also overcoming several disadvantages faced in other biological fluids such as easy collection, no diurnal variations (results not shown), less liability to external contamination, no need for vet personnel for sample collection, minimum or no sample pre-concentration or treatment, and ability for relatively longterm monitoring (e.g. few weeks) required for sample build-up.

On the other hand, the study of some important parameters that may influence the application of earwax as a diagnostic biological matrix in animals such as effect of grazing, diet, quantity and quality of earwax in the same breed, etc.) should be addressed in future studies by the authors.

In conclusion, we believe that combining advantages of metabolomics with non-invasive sampling using earwax could hold a great promise in pregnancy and lactation research keeping in mind the technical and economic challenges.

## Conclusion

Identification of changes of metabolism could aid in the determination of abnormal metabolic states and prediction of some metabolic disorders such as pregnancy toxemia and fatty liver which could be advantageous to producers. In this work, metabolic profiles obtained using earwax analysis as a non-invasive potential alternative biological matrix, were successfully used to predict pre- and post-parturient metabolic changes which, in the future could possibly be applied for diagnosis of metabolic diseases and assessment of nutritional status of sheep.
